# Correlation between the suppressor of cytokine signaling-1 and 3 and hepatitis B virus: possible roles in the resistance to interferon treatment

**DOI:** 10.1186/1743-422X-11-51

**Published:** 2014-03-17

**Authors:** Ling-yao Du, Yao-li Cui, En-qiang Chen, Xing Cheng, Li Liu, Hong Tang

**Affiliations:** 1 Center of Infectious Diseases, West China Hospital of Sichuan University, Chengdu 610041 China; 2 Division of Infectious Diseases, State Key Laboratory of Biotherapy, Sichuan University, Chengdu 610041, China

**Keywords:** SOCS, Chronic Hepatitis B, PEG-IFN α-2b

## Abstract

**Background:**

The suppressor of cytokine signaling family (SOCS) is an important negative regulator in the JAK-STAT signaling pathway. This study was designed to explore the correlation between SOCS-1, 2 and 3, Hepatitis B Virus (HBV) and interferon (IFN), and the relationship between SOCS and IFN therapeutic efficacy.

**Methods:**

Four types of mouse models were established. Mice were administered with HBV replicative plasmid pHBV4.1 and IFN inducer Poly IC (Group A), pHBV4.1 (Group B), Poly IC (Group C) and saline (Group D), respectively. Liver tissues were harvested from the mice and SOCS expression was determined. Meanwhile, patients with chronic hepatitis B (CHB) were treated with pegylated interferon α-2b for 24-48 weeks. Liver biopsy was collected and the baseline SOCS expression was determined. Serum assay was performed for efficacy evaluation and correlation analysis.

**Results:**

In animal studies, the expression level of SOCS-1 and 3 was found in the descending order of B, A, C and D. The difference between Group B and D suggested that HBV could induce SOCS. The difference between Group A and C suggested that HBV could still induce SOCS with up-regulated endogenous IFN. The difference between Group C and D suggested that ploy IC could induce SOCS, while the difference between Group B and A suggested that Poly IC might have a stronger inhibition effect for SOCS. There was no difference in SOCS-2 expression. In clinical studies, eight of twenty-four enrolled patients achieved either complete or partial therapeutic response. The expression of both SOCS-1 and 3 was higher in CHB patients than in normal controls. The baseline HBV-DNA level was positively correlated with SOCS-1 and 3. The age, viral genotype, HBVDNA, SOCS-1 and SOCS-3 were found to be related to IFN efficacy.

**Conclusion:**

HBV could induce both SOCS-1 and 3 expression regardless of endogenous IFN level. Elevated IFN could directly up-regulate SOCS-1 and 3 expression, but it could also indirectly down-regulate SOCS-1 and 3 expression by inhibiting HBV replication. HBV might play a more important role in the SOCS up-regulation than IFN, a possible reason why patients with high HBV viral load encounter poor efficacy of IFN treatment.

## Background

The antiviral therapy, immunomodulatory therapy and anti-inflammatory therapy are the most common treatments for patients with Chronic Hepatitis B (CHB) [1], among which the antiviral therapy with interferon (IFN) or nucleoside analogues (NA) is the key treatment [2,3]. INF-α has been widely accepted by the public for its use as an antiviral drug. However, there are still many unknown impact factors that affect its efficacy [4,5]. The therapeutic efficacy of INF is mainly achieved through the complicated "IFN System" where several signaling transduction pathways are activated by the binding of INF-α to INF-α receptor (INFAR). The pathway of Janus kinase-the signal transducer and activator of transcription (JAK-STAT) is one of the typical pathways [6]. In this pathway, INF-α binds to INFAR-I on the membrane to form a dimer, which activates Jak-1 and Tyk2, the signal transducers in the cytoplasm, to phosphorylate and activate STAT1 and STAT2. Activated STAT dimer translocates into the nucleus to bind the interferon-sensitive response elements (IFNSRE). Antiviral protein products are then induced to inhibit HBV-DNA transcription, promote HBV mRNA degradation and suppress HBV protein translation [7,8].

The suppressor of cytokine signaling (SOCS) is induced by cytokines and is an important negative regulatory factor in the JAK-STAT signaling pathway. So far, eight members of the SOCS family have been found, which include SOCS 1-7 and cytokine inducible Src Homology 2 (SH2) containing protein (CIS). They all consist of an amino-terminus, a SH2 domain in the middle, and a SOCS box in carboxy-terminus [9-11]. It has been demonstrated that SOCS protein could inhibit the activity of JAKs and compete with STAT2 to bind to the phosphorylated tyrosine residues in cytokine receptors through its SH2 domain. As a result, the phosphorylation of STATs is reduced. SOCS protein could also mediate the degradation pathways of the activated signaling proteins or bind to the cytoplasmic protein tyrosine phosphatase SHP2, thus inhibiting the signal transduction [12].

Recent studies have suggested that SOCS-1 and SOCS-3 are the negative regulators in the IFN signal transduction pathway in patients with chronic hepatitis C (HCV) infection. HCV core protein can induce SOCS-3 protein expression to reduce the therapeutic effect of IFN-α [13-15]. However, in CHB patients with IFN treatment, it’s unclear whether SOCS related elements impact INF efficacy in addition to HBV genotype and viral load. Therefore, further investigation is required to determine the correlation between SOCS expression, HBV and IFN therapeutic efficacy in CHB patients. In this study, mouse models of HBV replication were established to explore the interactions between SOCS expression and HBV. The expression of SOCS family in CHB patients during IFN treatment was also investigated to explore the correlation between the therapeutic efficacy of IFN and the expression of SOCS.

## Methods

### Animal studies

#### Study design

Twenty-four healthy BALB/c male mice at 6-9 weeks of age and 18-20 g of weight were purchased from Experimental Animal Center, Sichuan University, and divided into four groups. Mice in Group A were administered with the replicative HBV plasmid DNA pHBV4.1-DNA (10 μg pHBV4.1-DNA dissolved in saline equivalent to 8% mouse body weight) via the caudal vein using high-pressure injection. 24 hours after the injection, the mice were injected intraperitoneally with Poly IC at 200 μL/day from day 0 to day 3. Mice in Group B were administered with an equal dose of pHBV4.1-DNA via the caudal vein as in Group A, followed by intraperitoneal injection of saline at 200 μL/day from day 0 to day 3. Mice in Group C received the caudal vein high-pressure injection of an equal volume of saline and the intraperitoneal injection of Poly IC at 200 μL/day. Mice in Group D received both the caudal vein high-pressure and intraperitoneal injection of saline. Six hours after the last injection, these mice were euthanized and the liver tissues were harvested.

The plasmid DNA pHBV4.1-DNA we used here is an HBV replication competent plasmid. It contains 1.3 copies of the HBV genome of serotype ayw and is capable of HBV transcription, replication and expression both in vitro and vivo [16,17]. It’s kindly given by the Scripps Research Institute, La Jolla, CA, USA as a gift. The mouse model we established here was verified effective in our previous studies. It’s a transient HBV transfection model with viral replication and expression lasting for 10 days and peaking at day 3 and day 4. It fulfilled the applicable requirement in the study of HBV and related host factors [18].

#### Tissue sample analysis

Part of the mouse liver was fixed by formalin for immunohistochemical staining to detect the expression of SOCS proteins. Primary antibodies included anti-SOCS-1, anti-SOCS-2 (Santa Cruz) and anti-SOCS-3 (Abcam). PBS was used as the negative control for primary antibodies. Samples with known SOCS expression were used as the positive controls. As the SOCS proteins would be stained brown, positive sample was defined as those with stained particles present in the cytoplasm, or with stained cytoplasm. Two pathologists with no prior knowledge of the study were asked to score the tissue sections according the Axiotis score standard. The percentage of positive cells and intensity of positive staining scored as follows:

### Percentage score

0 = 0 ~ 10% positive cells;

1 = 11 ~ 25% positive cells;

2 = 26 ~ 50% positive cells;

3 = 51 ~ 75% positive cells;

4 = 76 ~ 100% positive cells.

### Intensity score

0 = no colour;

1 = yellow;

2 = brown;

3 = tan.

The sum of the percentage score and intensity score equaled the final score if there was no difference in two pathologists’ opinions. Or the mean of two sum scores would equal the final score [19].

The remaining liver tissue was put in liquid nitrogen immediately after the harvest and stored at -70°C. Tissues were ground into powder in liquid nitrogen. Total RNAs were extracted from the ground liver tissue and reversely transcribed into total cDNA by RT-PCR amplification. The sequences of mouse SOCS-1, SOCS-2, SOCS-3 and GAPDH genes were amplified from cDNA for semiquantitative analysis. Protein samples extracted from liver powder were analyzed using western blotting to evaluate the expression of SOCS proteins.

### Clinical studies

#### Study design

All human subjects enrolled should meet the criteria including qualified age from eighteen to sixty-five years old, free of antiviral and immunomodulating treatment in the past six months, negative pregnancy test in female in the past 24 hours and effective contraception in both sexes during the study. They should also have positive serum HBsAg for more than 24 weeks, elevated serum HBV-DNA at more than 5 × 10^5^copies/ml and twice elevated ALT between 2-10 ULN in the past 6 months. All subjects were provided with written informed consent.

The exclusion criteria included lactating women, decreased albumin at less than 3.5 g/L, extended prothrombin time for more than 4 s, elevated serum bilirubin at more than 34umol/L, decreased neutrophile granulocytes at less than 1.5 × 10^9^/L, decreased platelets at less than 9.0 × 10^12^/L, coinfection with other hepatitis viruses and accompaniment of diabetes, thyroid dysfunction, autoimmune diseases and psychological issues.

After the enrollment, all human subjects were subcutaneously administered with pegylated interferon α-2b (Peg-Intron™, Schering Plough, USA), with the dosage and duration varied based on the individual weight, and both personal choice and therapeutic response, respectively. The observation lasted for 48 weeks and had little impact on the conventional medical course. All subjects accepted physical examination, serum assay and liver biopsy before the treatment. At Week 12, 24 and 48 during the treatment only physical examination and serum assay were performed for therapeutic response evaluation.

### Sample analysis

The liver tissue from the human subjects was examined by immunohistochemistry to evaluate the expression of SOCS family. Normal liver tissue sample was obtained from the tissue bank of West China Hospital, SCU. As mentioned before, the SOCS proteins would be stained brown as well. Samples with stained particles in the cytoplasm were defined as positive samples. The expression level of SOCS were also scored by the two pathologists mentioned before.

The serum samples collected at each medical assessment were detected for HBV-DNA quantification (Lightcycler-480, Roche, Switzerland), serum makers of HBV infection (Alisei Quality System, RADIM, Italy), liver and kidney function (Modular EVO, Roche, Switzerland) and prothrombin time (Sysmex CA-7000 Systems, Sysmex, Japan). The human subjects also received routine blood and urine examination and electrocardiogram. According to the therapeutic efficacy, those with complete or partial virological, serological or biochemical responses were assigned into the response group, while the rest were assigned into the non-response group.

### Statistical analysis

Five different areas of each liver tissue section after immunohistochemical staining were scored and the RT-PCR and western blotting were repeated three times. The result of RT-PCR amplification was semi-quantified by a professional image analysis software Image Pro Plus 4.5 (Media Cybernetics Inc, Silver Spring, MD,USA). The outcome of western blotting was semi-quantified by Image J, a public domain Java image processing program inspired by NIH Image for the Macintosh. All data were used for statistical analysis. Measurement data were tested for normality and presented as mean ± standard deviation. Statistical analysis was performed by t test, χ^2^ test, and Wilcoxon and Spearman’s rank correlation test for measurement data, enumeration data, and ranked data, respectively. Logistic regression was used to determine the factors that would affect the efficacy of PEG-IFN treatment in the CHB patients. All statistical analysis was performed using Stata 10.0 and p < 0.05 was defined as significant difference.

### Ethics statement

This study was approved by both the Laboratory Animal ethics committee of Sichuan University and the West China Hospital Ethics Committee. Written informed consents were obtained from all patients before we carried out the liver biopsy.

## Results

### Animal studies

#### Expression of SOCS-1 in liver tissues

**Figure 1:**
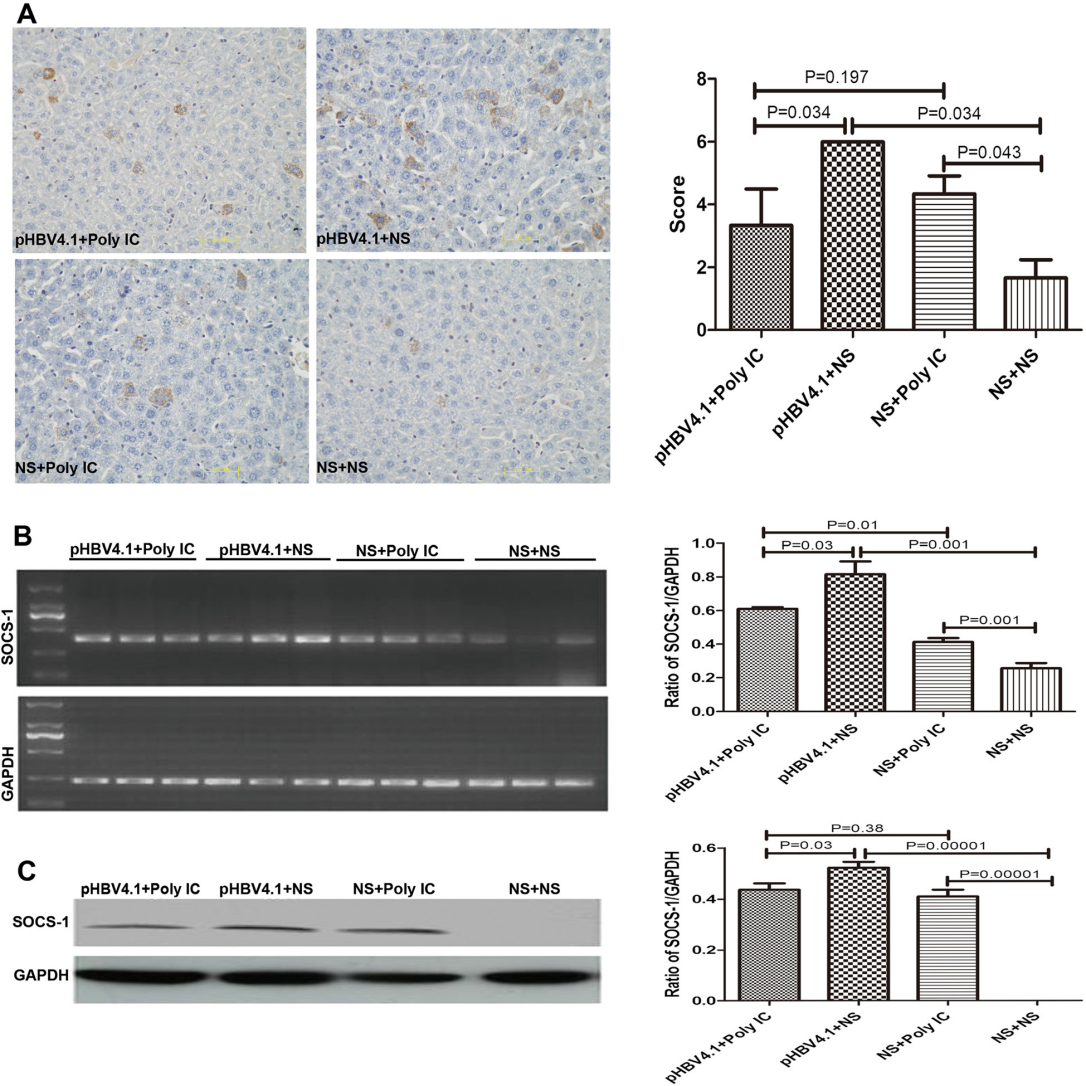
**The expression of SOCS-1 in mouse liver tissues. A**. Representative IHC images showing the expression of SOCS-1 in mouse liver tissues (×400. HE) and statistical diagram of score; **B**. Representative RT-PCR image of SOCS-1 mRNA in mouse liver tissues and statistical diagram of three times results quantified and expressed as a ratio of SOCS-1/GAPDH; **C**. Representative western blotting image of SOCS-1 proteins expression in mouse liver tissues and statistical diagram of three times results quantified and expressed as the ratio of SOCS-1/GAPDH.

In this study, four groups of mice were administered with HBV replicative plasmid pHBV4.1 followed with IFN inducer Poly IC (Group A), pHBV4.1 (Group B), Poly IC (Group C) and saline (Group D), respectively. SOCS-1 expression was found in all four groups with the SOCS-1 expression level in descending order from groups B, A, C, to D as shown by immunohistochemistry, RT-PCR, and western blotting (Figure 1). The expression of SOCS-1 in Group B was significantly higher than that in Group D, suggesting that HBV could induce the expression of SOCS-1. The expression difference between Group A and C suggested that HBV could still induce SOCS with up-regulated endogenous IFN. The expression in Group C was significantly higher than that in Group D, suggesting that after the direct up-regulation of endogenous IFN, SOCS-1 expression might be up-regulated via some negative feedback pathway. The expression in Group B was significantly higher than that in Group A, suggesting that Poly IC might not only mediate the up-regulation of SOCS-1 via increasing endogenous IFN level to inhibit HBV replication, but also have a stronger inhibiting effect than its inducing ability.

#### Expression of SOCS-2 in liver tissues

**Figure 2:**
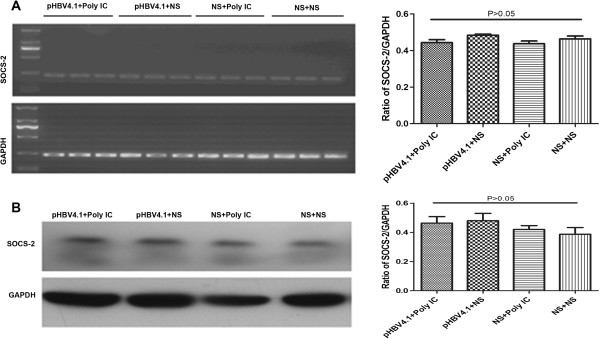
**The expression of SOCS-2 in mouse liver tissues. A**. Representative RT-PCR image of SOCS-2 mRNA in mouse liver tissues and statistical diagram of three times results quantified and expressed as a ratio of SOCS-2/GAPDH; **B**. Representative western blotting image of SOCS-2 proteins expression in mouse liver tissues and statistical diagram of three times results quantified and expressed as the ratio of SOCS-2/GAPDH.

There was no significant difference in SOCS-2 expression levels in all groups as determined by RT-PCR and western blotting (Figure 2), suggesting that the expression of SOSC-2 in mouse liver was affected by neither HBV nor Poly IC.

#### Expression of SOCS-3 in liver tissues

**Figure 3:**
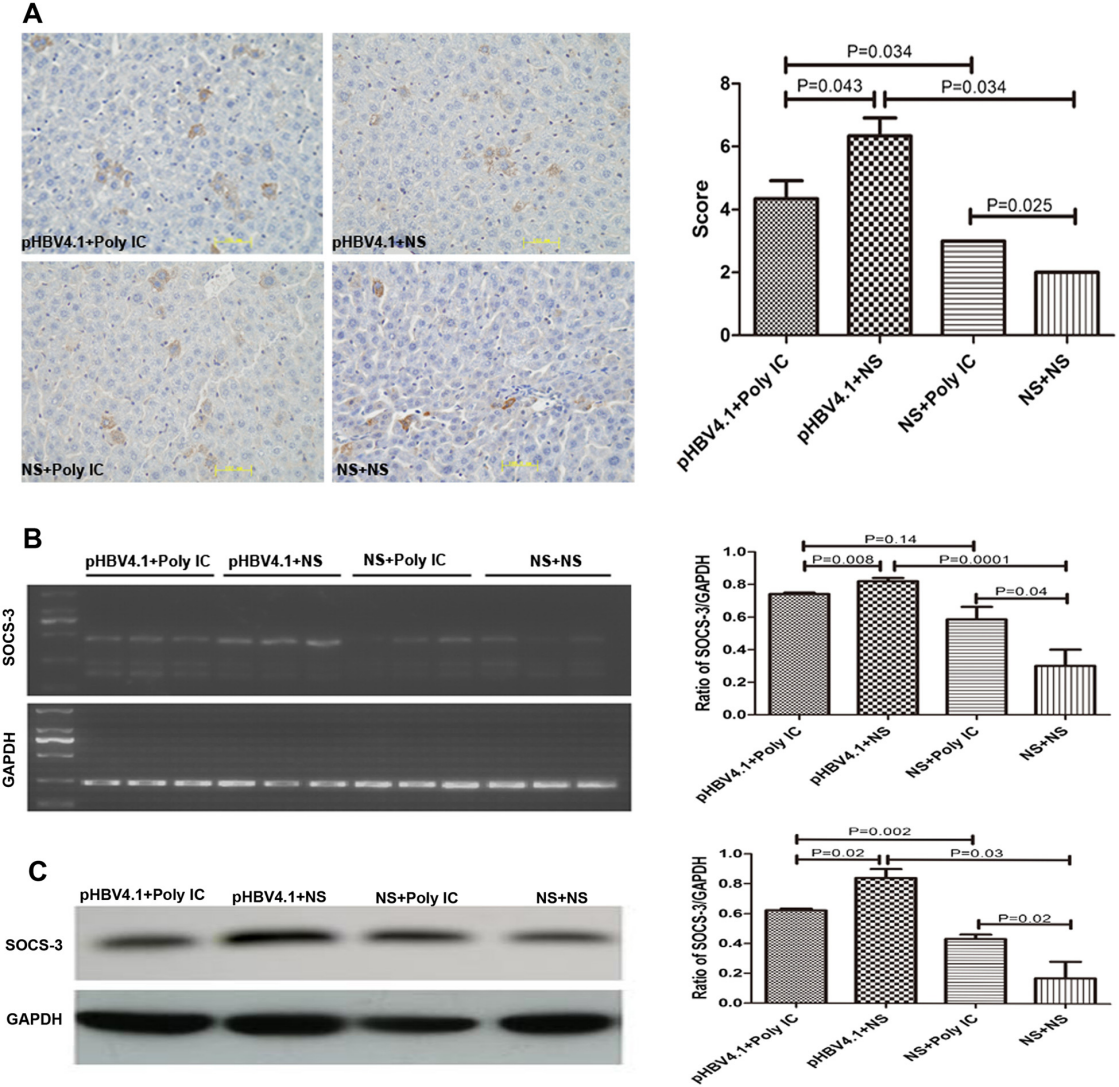
**The expression of SOCS-3 in mouse liver tissues. A**. Representative IHC images showing the expression of SOCS-3 in mouse liver tissues (×400. HE) and statistical diagram of score; **B**. Representative RT-PCR image of SOCS-3 mRNA in mouse liver tissues and statistical diagram of three times results quantified and expressed as a ratio of SOCS-3/GAPDH; **C**. Representative western blotting image of SOCS-3 proteins expression in mouse liver tissues and statistical diagram of three times results quantified and expressed as the ratio of SOCS-3/GAPDH.

Consistent with the expression of SOCS-1, SOCS-3 positive cells were found in all groups using immunohistochemistry. The expression level of SOCS-3 was in the descending order of B > A > C > D (Figure 3), same as that of SOCS-1. These results suggested that the expression of SOCS-3 and SOCS-1 was similarly mediated by HBV and Poly IC.

### Clinical studies

#### Subjects

Twenty-four subjects, including 17 female and 7 male, were enrolled for clinical observation. The average age was 25.54 years old. Detailed patient information was listed (Table 1).

**Table 1:** General information of the enrolled 24 CHB patients

	**Patients (n = 24)**
Age (years) (Mean ± SD)	25.54 ± 8.08
Gender (Male/Female)	17 / 7
Gene Type (A/B/C)	1 / 14 / 9
HBeAg (+/–)	24 / 0
HBeAb (+/–)	0 / 24
ALT (IU/L)	224.79 ± 95.82
AST (IU/L)	107.00 ± 62.45
ALB (g/L)	36.98 ± 2.21
TBIL (mg/dL)	18.50 ± 11.57
CHE (U/L)	5857.50 ± 623.08
INR for PT	2.26 ± 0.57
HBV DNA level (lg IU/ml)	17.17 ± 1.84

#### Therapeutic response to PEGINF α-2b in CHB patients

Fourteen patients (14/24) received 24 weeks’ treatment, and among them 5 achieved either complete or partial therapeutic response according to the judgmental criteria of the therapeutic responses in subjects. Among these 5 patients, 3 achieved HBeAg seroconversion. Another ten patients (10/24) received 48 weeks’ treatment, and among them 3 achieved HBeAg seroconversion. The 8 patients who achieved either complete or partial therapeutic response were assigned into the responder group, while the remaining 16 patients were assigned into the non-responder group (Table 2).

**Table 2:** The therapeutic response in CHB patients after treatment.

**Treatment course**	**Responders (N = 8)**		**Non-responders (N = 16)**	
	**HBeAg seroconversion**	**Non-HBeAg seroconversion**	**HBeAg seroconversion**	**Non-HBeAg seroconversion**
24 weeks	3	2	0	9
48 weeks	3	0	0	7

#### Expression of SOCS-1, 2 and 3 in the liver tissue of normal controls and CHB patients before treatment

**Figure 4:**
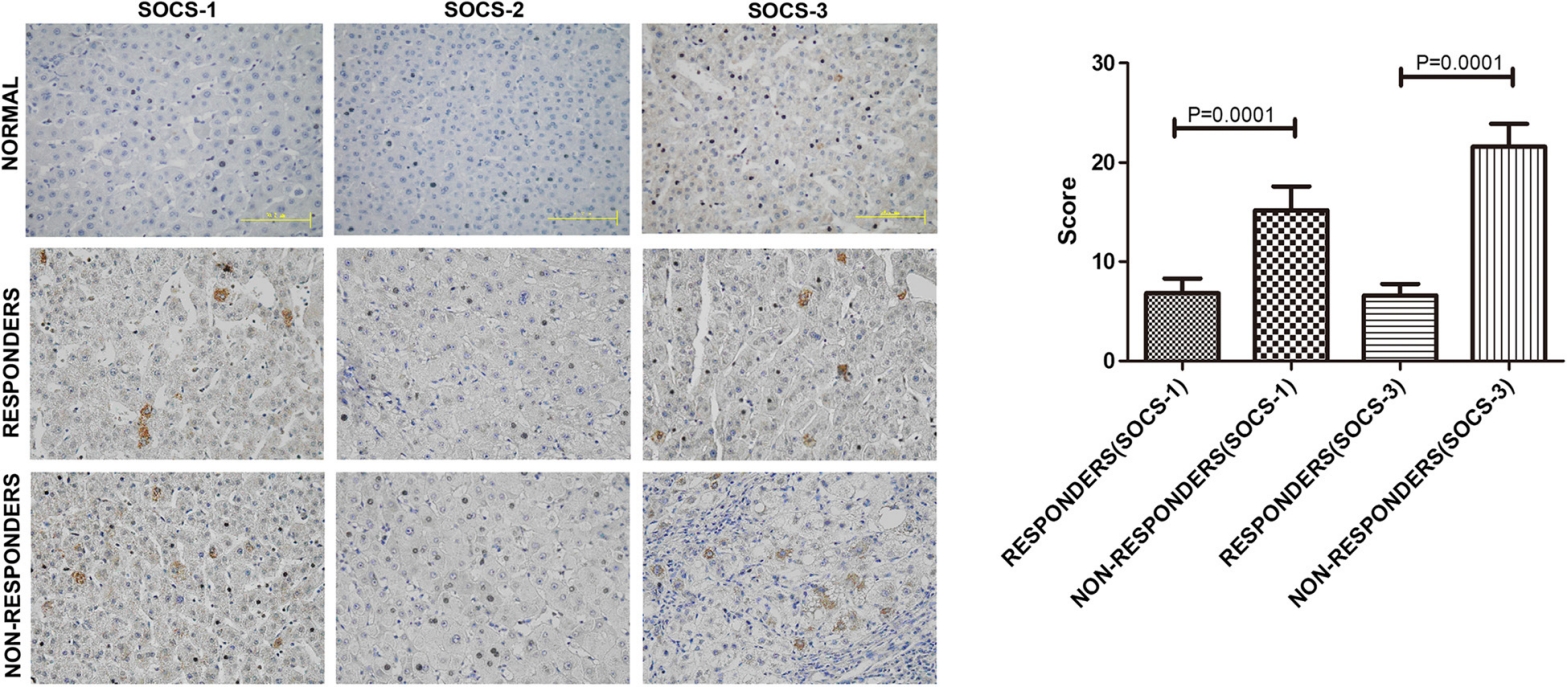
**The expression of SOCS-1, 2 and 3 proteins in patients before treatment.** The representative IHC images showing expression of SOCS-1, SOCS-2 and SOCS-3 proteins in all CHB patients before treatment and normal controls as well as the statistical diagram of score.

Both SOCS-1-positive and SOCS-3 positive cells were observed in the liver tissues of the CHB patients from both the responder and non-responder groups. SOCS-1 and SOCS-3 were mainly found in the cytoplasm of the hepatocytes. In contrast, only a trace of SCOS-3 was found in the liver tissues from the normal controls (Figure 4). In addition, there was a significant difference in the expression level of both SOCS-1 and SOCS-3 between the responder and non-responder groups before the treatment. The expression of SOCS-1 and SOCS-3 was higher in the non-responder group than in the responder group. SOCS-2 was not detected in any of the liver tissues examined.

#### Factors affecting the therapeutic efficacy

Four serum assays representing the hepatic function were performed before the treatment. Results were analyzed for correlation with SOCS-1 and SOCS-3 using Spearman Rank Correlation Test. Only the baseline HBV-DNA level was positively correlated with SOCS-1 and SOCS-3 (p < 0.05), while none of the ALT, AST or TBIL levels showed significant correlation with SOCS-1 or SOCS-3 (Table 3).

**Table 3:** The correlation between SOCS family, liver function and HBVDNA

		**SOCS-1**		**SOCS-3**	
**Item**	**Mean ± SD**	**Correlation**	***P *** **value**	**Correlation**	***P *** **value**
ALT (IU/L)	224.79 ± 95.82	-0.29	0.17	-0.33	0.11
AST (IU/L)	107.00 ± 62.45	-0.17	0.42	-0.20	0.36
TBIL (mg/dL)	20.10 ± 11.24	0.07	0.74	-0.17	0.44
HBV DNA level	17.17 ± 1.84	0.49	0.03	0.59	0.002

To investigate the factors which might affect the therapeutic efficacy of PEGINF α-2b, 15 possible factors including SOCS-1 and SOCS-3 expression level were chosen for the univariate analysis. Five factors showed significant differences between the responder and non-responder groups, suggesting that SOCS-1 and SOCS-3, in addition to the gender, viral genotype and HBV-DNA, might impact the therapeutic response (Table 4).

**Table 4:** **Univariate analysis of the factors affecting IFN**α**-2b therapeutic efficacy**

	**Responders (n = 8)**	**Non-responders (n = 16)**	**χ**^**2**^**/t/U**	**P values**
Age (years) (Mean ± SD)	23.75 ± 5.60	25.50 ± 9.45	-0.48	0.64
Gender (Male/Female)	7/1	6/10	–	0.03
Genotype (A/B/C)	0/6/2	1/3/12	7.29	0.03
HBeAg (+/–)	8/0	16/0	–	–
HBeAb (+/–)	0/8	0/16	–	–
ALT (IU/L)	251.50 ± 108.03	205.19 ± 94.44	-1.03	0.31
AST (IU/L)	106.63 ± 36.92	107.19 ± 73.09	-0.02	0.98
ALB (g/L)	37.95 ± 1.48	36.49 ± 2.39	1.57	0.13
TBIL (mg/dL)	24.27 ± 13.63	25.62 ± 9.59	-0.28	0.78
CHE (U/L)	6218.50 ± 477.86	6677.25 ± 620.29	-1.83	0.08
CREA (mg/dL)	72.11 ± 62.02	67.49 ± 9.55	0.30	0.77
INR for PT	2.20 ± 0.41	2.02 ± 0.50	0.88	0.39
HBV DNA level	15.34 ± 1.29	18.09 ± 1.32	-4.85	0.001
SOCS-1	5.13 ± 1.89	15.38 ± 3.65	-7.40	0.001
SOCS-3	6.75 ± 1.58	20.63 ± 3.24	-11.37	0.001

Logistic regression was used to determine the correlation between the 15 factors. Five variables including gender, viral genotype, HBV-DNA, SOCS-1 and SOCS-3 were admitted into the regression model (Table 5). To prove the sensitivity and specificity of the model, the receiver operating characteristic curve (ROC curve) and cut-off point were calculated. The area under the ROC curve in this logistic regression model was 82.68% and the cut-off point was 0.50, suggesting that the model was valid.

**Table 5:** **Multivariate analysis of the factors affecting IFN**α**-2b therapeutic efficacy**

**Prognostic factors**	**Regression coefficent**	**Standard error**	***Z *** **value**	***P *** **value**	**OR value**	**95% confidence interval**
Age	1.03	0.77	1.33	0.18	2.80	[-0.48~2.54]
Gender	0.32	0.55	0.58	0.03	1.37	[0.75~1.39]
Genotype (A/B/C)	0.96	0.27	3.51	0.04	2.62	[0.73~1.50]
HBeAg (+/–)	0.86	0.31	2.80	0.75	2.37	[-0.26~1.47]
HBeAb (+/–)	-0.80	0.27	-2.92	0.63	0.45	[-1.34~1.27]
ALT (IU/L)	-0.58	0.32	-1.82	0.07	0.56	[-1.21~0.05]
AST (IU/L)	-1.93	0.34	-5.65	0.96	0.14	[-2.60~1.26]
ALB (g/L)	0.17	0.77	0.22	0.83	1.62	[-1.35~1.68]
TBIL (mg/dL)	0.001	0.004	1.40	0.16	1.00	[1.00~1.24]
CHE (U/L)	2.18	1.23	1.78	0.08	8.91	[0.80-99.22]
CREA (mg/dL)	-0.04	0.03	-1.55	0.12	0.96	[0.91~1.01]
INR for PT	-0.003	0.001	-1.94	0.05	0.99	[0.99~1.00]
HBV-DNA	0.003	0.001	2.03	0.04	1.00	[1.00~1.01]
SOCS-1	-3.67	1.49	-2.47	0.03	0.03	[0.01~0.47]
SOCS-3	-2.50	1.24	-2.02	0.04	0.08	[0.01~0.93]
Cons	-0.11	4.07	-0.03	0.98	—	—

## Discussion

CHB patients usually encounter poor therapeutic response with IFN treatment. Therefore, we set to determine whether HBV might influence the efficacy via some negative cytokine regulators. The JAK/STAT pathway is the most important antiviral signaling transduction pathway of IFN, and SOCS proteins induced by cytokines are the key negative regulating proteins in the JAK/STAT pathway. By inhibiting the activity of JAK and competitively binding to the phosphorylated tyrosine residues in cytokine receptors, SOCS proteins reduce the phosphorylation of STATs. SOCS proteins also act as adaptor molecules to guide the active cell signals to biodegradation pathway [10]. Recently the SOCS-3 protein induced by HCV core protein has been shown to be important for HCV absconding from host INF [20,21]. However, there are only a few studies on the correlation between HBV and SOCS proteins, which focus on single HBV protein such as HBx protein. HBx has been shown to affect the JAK/STAT pathway in the transfected hepatocytes [22,23]. The subcellular mislocalization of the mutant HBx has been found to up-regulate STAT3 activation, resulting in STAT1 inhibition and SOCS-1 and SOCS-3 expression silencing. In the HBV-related hepatocellular carcinoma, the dysregulation of STAT/SOCS signaling is involved in the hepatocarcinogenesis [24-26].

It was found that SOCS-3 expression increased in liver specimens from patients with CHB and was positively correlated with the severity of inflammation. Same result was also demonstrated in the cell culture. However, the change of SOCS-1 level was not observed [27]. In our animal studies, the change of the two SOCS proteins was observed. The expression level of SOCS-1 and SOCS-3 proteins varied in different groups. The higher expression in Group B (pHBV4.1) as compared to Group D (NS) suggested that HBV induced the expression of SOCS-1 and SOCS-3 proteins. Poly IC is a well-known inducer of the endogenous IFN. Therefore, the higher expression of SOCS in Group A (pHBV4.1 + Poly IC) than in Group C (Poly IC) suggested that HBV could still induce SOCS expression with up-regulated endogenous IFN. Moreover, the expression level in Group C was higher than in Group D, suggesting that Poly IC might induce the up-regulation of the endogenous IFN, and then as a negative cytokine in IFN pathway, induce the expression of SOCS-1 and SCOS-3 via some negative feedback pathway. As HBV and Poly IC were both inducers, the expression level in Group A was supposed to be higher. However, IFN is an inhibitor for HBV. HBV inhibition-mediated decrease in SOCS-1 and SOCS-3 expression might be stronger than IFN up-regulation mediated increase in SOCS-1 and SOCS-3 expression, leading to the higher expression level in Group B. The expression of SOCS-2 protein was not significantly different among all groups, suggesting that HBV and Poly IC couldn’t affect SOCS-2 expression.

In previous clinical studies on the IFN treatment in CHB patients, the genotype and viral load of HBV were considered as the key indicators for INF therapeutic efficacy. Although many studies have focused on HBV, only a few focus on the host factors that might affect the INF therapeutic efficacy [4,28,29]. It has been reported previously that SOCS-1 and SOCS-3 proteins could down-regulate the INF therapeutic efficacy as negative regulators in the IFN signaling pathway in CHC patients, and the overexpression of SOCS-3 protein could be used for the prognosis of CHC patients with INF treatment. Therefore, it is possible that SOCS-1 and SOCS-3 proteins could similarly affect the therapeutic efficacy in CHB patients by negatively regulating IFN signaling transduction. Recently, it has been shown that plasmacytoid dendritic cells (pDCs) treated with HBsAg present a defect in INF-α secretion due to HBsAg-mediated upregulation of SOCS-1 expression [30], which might partially explain the HBV immune escape and persistent infection. In our clinical observation, under a natural HBV infection status, same results were obtained regarding the change of SOCS-1 and SOCS-3 as in the animal models. The higher expression of SOCS-1 and SOCS-3 in liver tissues of CHB patients compared to the normal controls again demonstrated the potential ability of HBV to induce SOCS-1 and SOCS-3. In addition, we also found higher expression of the two SOCS proteins in liver tissues in the responders as compared to the non-responders, suggesting that the efficacy of INF treatment might be impacted by the expression level of SOCS-1 and SOCS-3 proteins before the treatment. Negative correlation between the expression of SOCS proteins before treatment and the INF therapeutic efficacy was found using both one-way ANOVA and multivariate analysis. These results might explain why the patients with higher expression of SOCS-1 and SOCS-3 proteins in liver before treatment showed INF resistance, and the severity was consistent with the expression level.

HBx and HBsAg are able to change the expression of some SOCS proteins [24,30]. We showed that the most important medical indicator, HBVDNA, did interact with SOCS expression in CHB patients. In addition to the expression differences between the CHB patients and the normal controls, the expression of SOCS proteins also varied in patients with different viral load, which was positively correlated with the expression level of SOCS-1 and SOCS-3 proteins. The higher the viral load, the more expression of SOCS-1 and SOCS-3 in the liver tissues, and the more effective in their ability to block IFN-induced signaling pathway. Together with the results from animal studies, our data explained why the viral load is the most important indicator for IFN therapeutic efficacy predication. HBV induced the expression of SOCS proteins, which then inhibited IFN signaling transduction pathway. As a result, IFN antiviral efficacy was decreased. Thus, these results provided the theoretical foundation for studying the relationship between SOCS proteins in CHB patients before treatment and IFN therapeutic efficacy, and explained why HBV appeared to play a more important role than IFN in SOCS up-regulation. However, since the transfection efficacy of hydrodynamic delivery in this mouse model in vivo was too low, our findings need to be confirmed with further investigations.

In conclusion, HBV could induce SOCS-1 and SOCS-3 expression regardless of the level of the endogenous IFN. The elevated IFN would directly up-regulate SOCS-1 and SOCS-3 via some negative feedback pathway, and indirectly down-regulate SOCS-1 and SOCS-3 by inhibiting HBV. However, HBV played a more important role than IFN in SOCS up-regulation, a possible reason why patients with high HBV viral load would encounter poor efficacy in IFN treatment. HBV induced SOCS proteins, which could negatively regulate IFN signaling to decrease IFN therapeutic efficacy, making the expression of SOCS-1 and SOCS-3 proteins in liver before treatment an important impact factor in IFN treatment for CHB. SOCS genes can be the promising candidates in gene therapy and drug development. For example, adenovirus vector expressing the SOCS proteins can be used to treat rheumatic arthritis [31]. Moreover, RNA interference and antisense nucleic acid could also be used to down-regulate the SOCS expression, and some molecular compound could be used to enhance or block its inhibition of the cytokine signaling transduction [32]. New drugs for the JAK-STATs pathway can be developed if detailed mechanism by which SOCS proteins function under physiological and pathological condition is understood. Anti-cytokine and anti-SOCS proteins therapies might be the alternative opportunities for patients with IFN resistance.

## Competing interests

The contents are solely the responsibility of the authors and do not necessarily represent the views of the funding source. The authors declare that they have no competing interests.

## Authors’ contributions

TH conceived the study, provided fund support and revised the manuscript critically for important intellectual content. DLY, CYL, CEQ, CX and LL made substantial contributions to experiment, clinical studies and data analysis. DLY and CYL participated in interpretation of data and manuscript preparation. DLY draft the manuscript and revised it according to all author’s opinions. All authors have read and approved the final manuscript.
